# Whole-genome optical mapping reveals a mis-assembly between two rRNA operons of *Corynebacterium pseudotuberculosis* strain 1002

**DOI:** 10.1186/s12864-016-2673-7

**Published:** 2016-04-30

**Authors:** Diego César Batista Mariano, Thiago de Jesus Sousa, Felipe Luiz Pereira, Flávia Aburjaile, Debmalya Barh, Flávia Rocha, Anne Cybelle Pinto, Syed Shah Hassan, Tessália Diniz Luerce Saraiva, Fernanda Alves Dorella, Alex Fiorini de Carvalho, Carlos Augusto Gomes Leal, Henrique César Pereira Figueiredo, Artur Silva, Rommel Thiago Jucá Ramos, Vasco Ariston Carvalho Azevedo

**Affiliations:** Laboratory of Cellular and Molecular Genetics, Department of General Biology, Institute of Biological Sciences, Federal University of Minas Gerais, CEP 31270-901 Belo Horizonte, Minas Gerais Brazil; National Reference Laboratory for Aquatic Animal Diseases of Ministry of Fisheries and Aquaculture, Federal University of Minas Gerais, CEP 31270-901 Belo Horizonte, Minas Gerais Brazil; Centre for Genomics and Applied Gene Technology, Institute of Integrative Omics and Applied Biotechnology (IIOAB), Nonakuri, Purba Medinipur, WB 721172 India; Institute of Biological Sciences, Federal University of Pará, Belém, Pará Brazil

**Keywords:** Genomics, Sequencing, Optical mapping, Mis-assembly

## Abstract

**Background:**

Studies have detected mis-assemblies in genomes of the species *Corynebacterium pseudotuberculosis*. These new discover have been possible due to the evolution of the Next-Generation Sequencing platforms, which have provided sequencing with accuracy and reduced costs. In addition, the improving of techniques for construction of high accuracy genomic maps, for example, Whole-genome mapping (WGM) (OpGen Inc), have allow high-resolution assembly that can detect large rearrangements.

**Results:**

In this work, we present the resequencing of *Corynebacterium pseudotuberculosis* strain 1002 (Cp1002). Cp1002 was the first strain of this species sequenced in Brazil, and its genome has been used as model for several studies *in silico* of caseous lymphadenitis disease. The sequencing was performed using the platform Ion PGM and fragment library (200 bp kit). A restriction map was constructed, using the technique of WGM with the enzyme *Kpn*I. After the new assembly process, using WGM as scaffolder, we detected a large inversion with size bigger than one-half of genome. A specific analysis using BLAST and NR database shows that the inversion occurs between two homology RNA ribosomal regions.

**Conclusion:**

In conclusion, the results showed by WGM could be used to detect mismatches in assemblies, providing genomic maps with high resolution and allow assemblies with more accuracy and completeness. The new assembly of *C. pseudotuberculosis* was deposited in GenBank under the accession no. CP012837.

**Electronic supplementary material:**

The online version of this article (doi:10.1186/s12864-016-2673-7) contains supplementary material, which is available to authorized users.

## Background

*Corynebacterium pseudotuberculosis* (*Cp*) is a Gram-positive, pleomorphic, facultative intracellular pathogenic bacteria that belongs to the group *Corynebacterium*, *Mycobacterium*, *Nocardia* and *Rhodococcus* (CMNR) [1]. *Cp* can be classified into two biovars: *equi* and *ovis*. Biovar *equi* is characterized by its capacity to nitrate-reductase production, while the biovar *ovis*, cannot [2]. Genomic plasticity analysis using 15 *Cp* strains demonstrates that the group of strains belonging to the *ovis* biovar are highly similar [3]. *Cp* is the etiological agent of the caseous lymphadenitis (CLA) disease, that affects mainly sheep and goat causing huge economic losses by affecting meet and wool production [4, 5]. It is also capable to cause diseases in cattle and humans. However, so far there is no proper diagnosis method or effective treatment available for *Cp* infection.

With the advent of next-generation sequencing (NGS) platforms [6–8], so far 37 *Cp* genomes have been completely sequenced of which Cp1002 is the first sequenced genome [3, 9–14]. Sequencing of several new strains are ongoing in our laboratory.

Recently the Cp31 strain that was originally sequenced using the SOLiD v3 platform and mate-pair library [9], was re-sequenced using Ion PGM platform [15]. This new sequencing discovered a new ~91 Kbp fragment in the Cp31 genome that is not present in NCBI. Therefore, there are possibilities that some of the available *Cp* genomes in NCBI may be incomplete and warns resequencing, reassembly, and minimization or closing gaps.

Due to the presence of highly repetitive regions that code for phage sequences, transposons, plasmid, and ribosomal RNA (rRNA) [16] in genomes and lack of good assemble software, finishing of assemblies is most critical step in genome assembly process [17]. Several strategies have been used to perform the scaffold based assemble process, for example: (i) scaffolding by reference, (ii) scaffolding by mate-pair libraries, or (iii) scaffolding by optical maps.

In the reference strategy, the contigs are oriented and positioned based on similar regions in a reference genome. This is a cost effective and a totally *in silico* method that can be executed through scaffolding software such as CONTIGuator [18] or Mauve [19], in addition to closing gaps software, like MapRepeat [20]. However, this strategy is not able to detect large sequence modifications, *e.g.*, large inversions detected between operons rRNA [21] or large chromosomal rearrangement [22] among others. The scaffolding by mate-pair libraries uses the distance of paired reads present in the contigs extremities to detect their orders. SSPACE [23] and GapFiller [24] like software can perform scaffolding and gap closing using paired data. The typical values for paired distances are 3 Kbp, 6 Kbp, 8 Kbp or 20 Kbp. However, if the length of the repetitive regions is bigger than the paired reads distance, the software cannot perform the scaffolding process [25].

On the other hand, whole-genome mapping (WGM), also known as optical mapping, uses images of unique DNA molecules immobilized in a polarized glass surface. The molecules are digested *in situ* by restriction enzymes, fragments sizes are calculated, and the high-resolution physical restriction map are used to determine the fragments order [26, 27]. Thus, optical mapping is considered one of the most accurate techniques to perform contigs scaffolding and it has been used to finishing several bacterial genomes [28]. The WGM technique uses Argus system (OpGen Inc, Gaithersburg, MD) that can be divided into four steps: (i) Extraction of chromosomal DNA, (ii) immobilization and *in situ* restriction digestion, (iii) image capture and measurement, and (iv) map assembly and analysis [26].

Recently, optical mapping has been largely used with success to detect genetic inversions in bacterial genomes. For example, WGM was used to detect a large genetic inversion between two Methicillin-resistant *Staphylococcus aureus* strains [29]. In a long-term evolution experiment, WGM was combined with genome sequencing (WGS) and PCR to analyze rearrangements in twelve *Escherichia coli* populations propagated in a glucose-limited environment for over 25 years [22]. In this experiment, they detected 19 inversions where three inversions found to have sizes larger than one-half of the chromosome. Thus, WGM can be considered to detect large rearrangements and mismatches in assemblies.

### *Corynebacterium pseudotuberculosis* strain 1002

*Corynebacterium pseudotuberculosis* strain 1002 (Cp1002) was isolated from a *Caprine caseosus* in Curaça county, state of Bahia (Brazil) in 1971 [30]. Cp1002 was the first strain of this species sequenced in Brazil and its genome is used as a model for several studies of caseous lymphadenitis. Thus, this strain is considered to be representative for the *ovis* biovar and important for caseous lymphadenitis researches in Brazil.

The first sequencing of Cp1002 was performed using 454 Roche and Sanger that showed a circular genome with ~2.35 Mbp, G + C content of 52.2 %, 12 rRNA, 48 tRNA, 2,095 CDS, and 47 pseudogenes [13]. To finish the Cp1002 assembly, it was used the genetic order of *Corynebacterium* species with high similarity [13]. None experimental strategy was used to contigs scaffolding. Therefore, it is possible that mis-assemblies remained in the submitted genome of Cp1002 available in NCBI. Because of its importance in studies of caseous lymphadenitis, and after the results obtained previous studies [15], we consider Cp1002 as the candidate for a new sequencing in order to detect possible mis-assemblies.

In this work, we perform a resequencing of Cp1002 using the platform Ion PGM. We also construct a restriction map using the WGM technique (OpGen Inc, Gaithersburg, MD), and new assembly and annotation are performed. We also compared the newly obtained genome sequence with the first genome available at NCBI.

## Methods

### Strain and DNA isolation

Cp1002 was grown in brain-heart-infusion (BHI-HiMedia Laboratories Pvt. Ltd., India) at 37 °C under rotation. Extraction of chromosomal DNA was performed using 30 mL of 48–72 h culture of *C. pseudotuberculosis*, centrifuged at 4 °C and 4000 rpm for 15 minutes. Re-suspension of cell pellets was done in 600 μL Tris/EDTA/NaCl [10 mM Tris/HCl (pH 7.0), 10 mM EDTA (pH 8.0), and 300 mM NaCl], and transferred to tubes with beads for cell lysis using Precellys (2 cycles of 15 seconds at 6500 rpm with 30 seconds between them). Purification of DNA with phenol/chloroform/isoamyl alcohol (25:24:1) was followed by precipitation with ethanol/NaCl/glycogen (2.5 v, 10 % NaCl and 1 % glycogen). The DNA was re-suspended in 30 μL MilliQ water, the concentration was determined by spectrophotometer, and the DNA was visualized using 1 % agarose gel electrophoresis.

### Optical mapping

First, the DNA was extracted and isolated using Argus Sample Preparation Kit and Agencourt Genfind v2 DNA Isolation Kit. The DNA was immobilized and digested *in situ* in a MapCard Processor using the restriction enzyme (*Kpn*I). Thereafter, the molecules were imaged by fluorescence microscopy, and processed to detect restriction sites using the image acquisition software of Argus WGM system (OpGen Inc). Lastly, the Argus assembly software (OpGen Inc) was used to calculate a consensus of a restriction map and Argus MapSolver™ software (OpGen Inc, Gaithersburg, MD) was employed to import the DNA sequence and converted to *in silico* data.

### Sequencing, assembly and annotation

The genome of Cp1002 was sequenced using Ion Torrent PGM System with 200 bp sequencing kit. The analysis of quality of the reads was performed using the FastQC software (http://www.bioinformatics.babraham.ac.uk/projects/fastqc) and showed a Phred value, in most cases, greater than 20. Hence, it was not applied trimming or quality steps to raw reads before assembly. The *de novo* assembly was performed using Mira 3.9.18 [31] applying the parameters “-GE:not = 16 IONTOR_SETTINGS -AS:mrpc = 100”. The scaffolding and gap closing were performed with SIMBA software (http://ufmg-simba.sourceforge.net) using the report generated by the software MapSolver™ (http://opgen.com/genomic-services/softwares/mapsolver) as reference to the scaffolder. The finishing of the genome was done using CLC Genomics Workbench 7.0 (Qiagen, USA) and the Website BLAST (http://blast.ncbi.nlm.nih.gov/Blast.cgi). The annotation was performed using in-house scripts to fetch the annotations of a manually curated *C. pseudotuberculosis* genome annotation database obtained in the UniProt database (http://uniprot.org). Finally, the pseudogenes were curated manually using the Artemis software [32] and the UniProt database.

### Comparing assemblies

To validate and to compare the new assembly (we called as Cp1002B) with the old genome of *C. pseudotuberculosis* 1002 available at NCBI (NC_017300) (we termed as Cp1002A), we performed the alignment between the experimental restriction map (obtained by WGM) of *C. pseudotuberculosis* 1002 with Cp1002B and with Cp1002A using MapSolver™ software (default parameters were used).

Thereafter, we used a modified version of the software CONTIGuator [18] to generate a syntenic comparison between Cp1002A and Cp1002B. For this comparison, we used the complete genome in a FASTA format for both the assemblies. Additionally, the annotation file (GenBank file) of Cp1002, the Website BLAST and NR database were used to detect repetitive regions that could be involved in possible genomic rearrangements.

## Results

### *De novo* assembly and annotation

The new assembly Cp1002B on Mira showed 9 contigs through 731,481 reads, with a N50 value of 402,955 bp and a deep coverage of ~58-fold (Table 1). The genome represents a circular chromosome of 2,335,107 bp, 52.2 % of G + C content, 12 rRNA, 48 tRNA, 2,071 CDS, and 43 pseudogenes.

**Table 1:** Statistics of the *C. pseudotuberculosis* 1002 new assembly

Assembler	Mira 3.9.18
Reads assembled	731,481
Contigs	9
Shortest contig	4,133
Largest contig	542,891
N50	402,955
N90	218,254
N95	147,989
Total coverage	58.63

### Comparison between assemblies of Cp1002

**Fig. 1:**
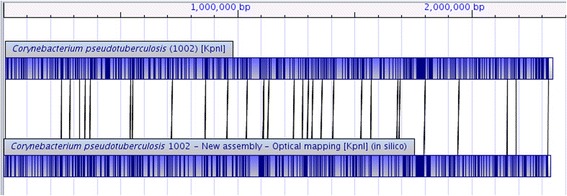
Alignment between the restriction map of *C. pseudotuberculosis* 1002 (*above*) and the *in silico* map of the new assembly of *C. pseudotuberculosis* 1002 (*below*). Both restriction maps were generated using the restriction enzyme *Kpn*I. The alignment shows a high similarity between the two restriction maps, indicating a high probability of a correct assembly

**Fig. 2:**
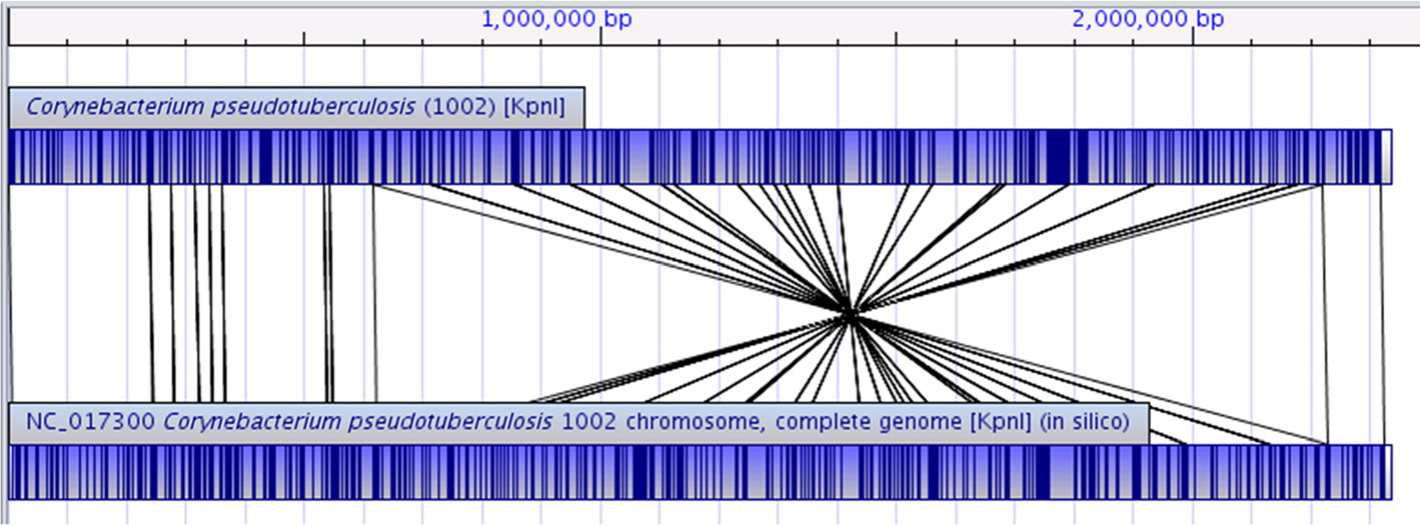
Alignment between the restriction map of *C. pseudotuberculosis* 1002 (*above*) and the *in silico* map of the complete genome of *C. pseudotuberculosis* 1002 (NC_017300) obtained from NCBI database (*below*). Both the restriction maps were generated using the restriction enzyme *Kpn*I. The alignment shows a large inversion between the two restriction maps. A detailed analysis using CLC Genomics Workbench 7, BLAST and NR database shows that the inversion occurs between two rRNA regions

The alignment between the experimental restriction map of Cp1002 (obtained by WGM) and the *in silico* restriction map of Cp1002B (obtained by MapSolver™) shows that the new assembly presents a high accuracy (Fig. 1). On the other side, the alignment between the experimental restriction map of Cp1002 and the *in silico* restriction map of Cp1002A shows a large inversion with a size larger than one-half of the genome (Fig. 2).

**Fig. 3:**
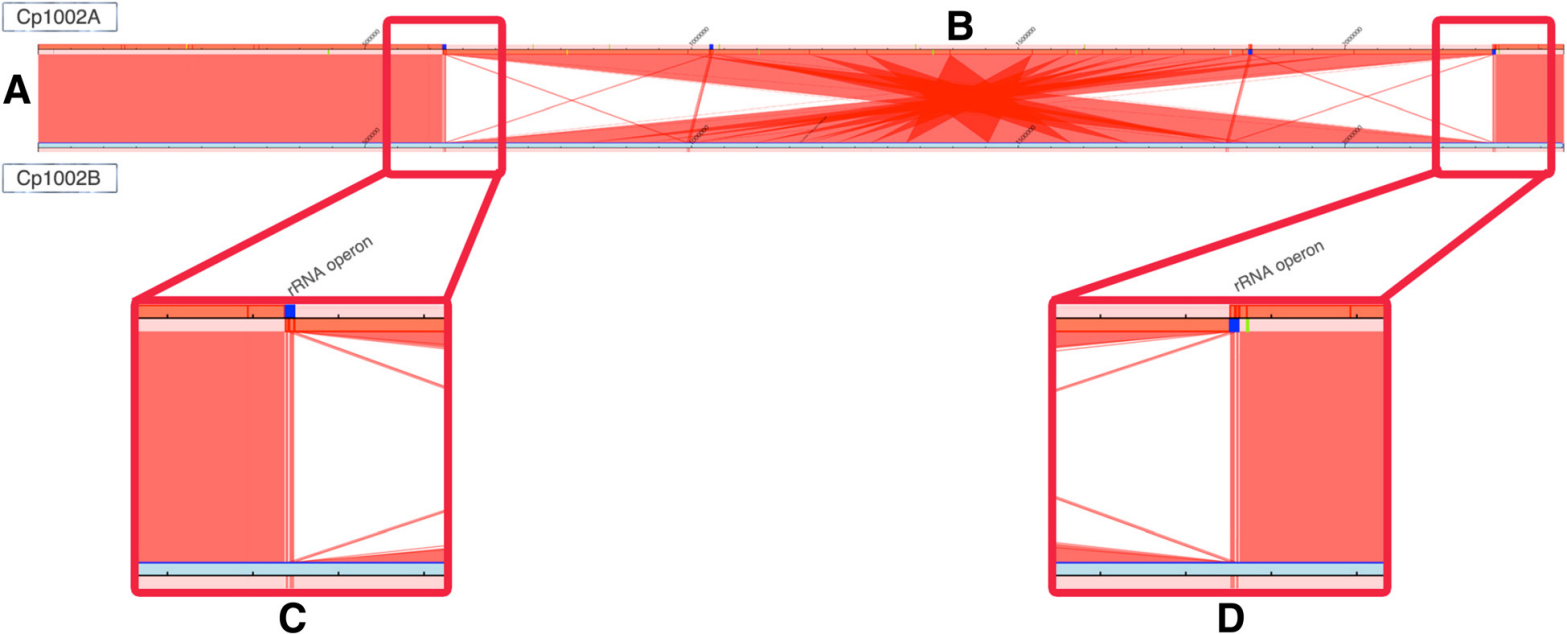
Syntenic comparison between the first assembly (Cp1002A) and the new assembly (Cp1002B). **a** The genome of Cp1002A is showed above, while the genome of Cp1002B in shown below. Red lines linking the line above and the line below indicate syntenic regions. The annotation of Cp1002A was used to insert color targets in the graph that detect repetitive regions: blue for rRNA operons, light blue for transposons, yellow for plasmids and green for phages. **b** The genomes are highly similar, except by a genetic inversion larger than 1 Mbp between two rRNA operons. **c** rRNA operon in the left side of the genetic inversion. It is possible to detect a change in the sense strand after the rRNA operon that indicates an inversion. **d** rRNA operon in the right side of the inversion sequence

The syntenic comparison between Cp1002A and Cp1002B (Fig. 3) shows a genetic inversion that occurs between two regions encoding ribosomal RNA. The inversion occurs between the first rRNA operon (Fig. 3c) and the last rRNA operon (Fig. 3d), both highlighted in blue color in the figures.

## Discussion

Our results showed that, in the new assembly, the number of CDS and pseudogenes are less in number as compared to the first assembly (Table 2). However, we believe that the new annotations are more accurate since bigger and improved databases are used. For instance, in Cp1002A we detected 592 CDS as hypothetical proteins, with an average length of 617 bp. However, in Cp1002B we detected 551 hypothetical proteins, with an average length of 632 bp; thus improving the annotation. In some cases, we observed that two small hypothetical proteins join to form one large hypothetical protein. The results also showed that there is only 6 bp difference between these two assembled genomes Cp1002A and Cp1002B. Although, this value can be considered insignificant, this difference can be due to the homopolymer errors undetected in the manual frameshift curation.

**Table 2:** Comparison between the assemblies of *C. pseudotuberculosis* 1002: Cp1002A (first assembly) and Cp1002B (new assembly)

	Cp1002A	Cp1002B
Genome length	2,335,113 bp	2,335,107 bp
CDS	2,095	2,071
Hypothetical proteins	592	551
Pseudogenes	47	43
Depth coverage	31x	58x
GC %	52.2 %	52.2 %
rRNAs	12	12
tRNAs	48	48

Previously, it was predicted that the Cp1002 genome presented high similarity in genomic architecture, gene content and genetic order when compared to other *Corynebacterium* species [13]. Indeed, the assembly of Cp1002A was performed using reference-based assemblies techniques with short reads as well as other *Cp* strains [14]. The large inversion detected here is a mis-assembly caused by the limitations of the reference-based assembly strategies. Although genomes of the same specie tend to show high synteny, reference-based strategies cannot detect large inversions, as the mis-assembly detected in this work. Mis-assemblies in *Cp* genomes have been detected previously using mate-pair libraries [15], however it is the first time that WGM was used to correct *Cp* genome assemblies. The WGM technique is efficient to provide high accurate assemblies [22, 28, 29], and in this work, it was important to correct the assembly of Cp1002.

Furthermore, we detected a large inversion between two operons that encodes rRNA. The genome of Cp1002A presents a high synteny with other *Cp* strains [13]. However, Cp1002B shows a large inversion. Occurrences of large inversions are reported in several bacterial species [21, 22, 29]. Before the age of modern techniques for constructions of optical mapping, it was established the genome map of *Salmonella paratyphi* A using four endonucleases, *XbaI*, *I-CeuI*, *AvrII* (*Bln*I), and *SpeI* to generate fragments that could be compared [21]. They also compare the results with maps of other *Salmonella* species, and detect an inversion of half the genome between rRNA operons *rrnH* and *rrnG*. They postulated that the presence of this inversion is due to homologous recombination between the ribosomal genes. Another work proposed that the mechanism of producing chromosomal rearrangements is recombinational exchanges between homologous sequences, as found in ribosomal operon, similar to our observation here [33]. The large inversion detected between two rRNA operons in Cp1002 is not reported in *Cp* genome strains belong to *ovis* biovar.

## Conclusions

Our new assembly (GenBank accession no. CP012837) was performed through a *de novo* strategy validated by experimental evidence (WGM), while the older assembly was performed by reference strategy. Thus, the new assembly corrected a large mis-assemble in Cp1002 genome that was not detected in the previous sequencing and assembly projects. Our optical mapping detected a large inversion between two rRNA operons in *Corynebacterium pseudotuberculosis* strain 1002. Inversion in *Cp* genome strains belong to *ovis* biovar are not reported so far but may be detected if we use WGM technique. However, the real effects of such major changes in the bacterial DNA need further evaluation.

## Ethics approval and consent to participate

Not applicable.

## Consent for publication

Not applicable.

## Availability of data and materials

The genome sequence for *C. pseudotuberculosis* 1002 (Cp1002B) has been deposited in the GenBank database (accession no. CP012837).

The WGM dataset used to the Cp1002B sequence placements by MapSolver™ is included within the article (Additional file 1).

## Additional file

Additional file 1:
*C. pseudotuberculosis* 1002 sequence placement. This XML file contains the restriction site positions used to the sequence placements by MapSolver™. (XML 88 kb)

## Abbreviations

CLA: caseous lymphadenitis
CDS: coding sequence
Cp: *Corynebacterium pseudotuberculosis*
Cp1002: *Corynebacterium pseudotuberculosis* strain 1002
Cp1002A: *Corynebacterium pseudotuberculosis* strain 1002 (first assembly)
Cp1002B: *Corynebacterium pseudotuberculosis* strain 1002 (new assembly)
Cp31: *Corynebacterium pseudotuberculosis* strain 31
NCBI: National Center for Biotechnology Information
PCR: polymerase chain reaction
WGM: whole-genome mapping
WGS: whole-genome sequencing
